# Prohibitin 1/2 mediates Dengue-3 entry into human neuroblastoma (SH-SY5Y) and microglia (CHME-3) cells

**DOI:** 10.1186/s12929-020-00639-w

**Published:** 2020-04-19

**Authors:** Amita Sharma, Ravi Vasanthapuram, Manjunatha M Venkataswamy, Anita Desai

**Affiliations:** Department of Neurovirology, National Institute of Mental Health and Neurosciences, 560029, Bengaluru, India

**Keywords:** Dengue virus serotype-3, Neural cells, SH-SY5Y, CHME-3 cells, Receptor/ interacting proteins, Prohibitin

## Abstract

**Background:**

Very few studies have identified receptor molecules for dengue virus (DENV) on neural cells. This study was designed to identify putative receptor/(s) involved in entry of DENV-3 in human neural cells of various lineages; neuronal-SH-SY5Y, astroglial-U-87 MG and microglial-CHME-3 cells.

**Result:**

Virus overlay protein binding assay, LC-MS/MS and SEQUEST identified prohibitin1/2 (PHB1/2) as interacting proteins on SH-SY5Y, CHME-3, and U-87 MG cells. Infection inhibition and siRNA assays confirmed the role of PHB1/2 in the entry of DENV-3 into SH-SY5Y and CHME-3 cells but not in U-87 MG cells. Indirect immunofluorescence and flow-cytometry demonstrated the presence of PHB1/2 on the surface of SH-SY5Y and CHME-3 cells. Co-immunoprecipitation and Western blot, as well as double labelling, reconfirmed the interaction between PHB1/2 and DENV-3 EDIII protein.

**Conclusion:**

These observations together for the first time indicate that PHB1/2 may serve as a putative receptor for DENV-3 in SH-SY5Y and CHME-3 cells. The study provided insights into DENV-3 and neural cell interactions.

## Background

Dengue virus, a member of the family Flaviviridae is an arthropod-borne virus that can cause a febrile illness (dengue fever, DF) in humans [1] and is occasionally associated with severe bleeding (dengue hemorrhagic fever, DHF) and hypovolemic shock (dengue shock syndrome, DSS). Although dengue is not primarily classified as a neurotropic virus, recent reports have recognized CNS manifestations in dengue virus-infected cases [2–4]. Increased frequency of these reports suggests expanded tropism of the virus. However, the precise molecular events involved in the neurotropism of DENV have not yet been investigated.

Virus binding to susceptible target cells is the first event required for productive infection. Identification of cellular receptors has been a stimulating field as many viruses exhibit a wide host range comprising of vertebrates and invertebrates [5]. The host range of a virus defines both the type of tissue that the virus infects and the animal species in which it multiplies [6]. Many viruses exploit multiple host cell surface attachment molecules sequentially or in a cell type-specific manner and co-receptor may also be involved [7].

Several molecules have been reported to be implicated in binding and entry into the host cells, only a few of these have been postulated to play a role in DENV-3 infection. In Vero and BHK cells, binding and entry of DENV-2 requires the presence of a highly sulphated form of heparin [8]. An earlier study suggested that a 65 kDa membrane protein may be involved in the binding of DENV-2 to the human neuroblastoma (SK-N-SH) cells [9] while another study identified HSP70/ HSP90 as a receptor on SK-SY5Y (human neuroblastoma) cells [10]. In case of DENV-3, receptors have been identified on a number of non-neural cells [11–14]. Mannose receptors have been identified on macrophage cell lines [12] while; a high-affinity laminin receptor was identified on porcine kidney PS clone D cells [11]. Although these studies have contributed to new knowledge, they have not addressed the cell surface proteins present on different types of neural cells (neurons, astroglia, and microglia) that interact and facilitate entry of DENV-3. This study was thus designed to identify and characterize cell surface proteins that could serve as receptors for entry of DENV-3 in neural cells.

## Methods

### Virus and cell lines

DENV-3 (16562) kindly provided by Dr. Lance Turtle (University of Liverpool, UK) was used in this study. DENV-3 was propagated in *Aedes albopictus* C6/36 cells grown in Eagle’s minimal essential medium (MEM- Gibco, USA) supplemented with 2 mM l-glutamine (Sigma Aldrich, USA) and 10% fetal bovine serum (FBS; Gibco, USA), at 28 °C. The human neuroblastoma (SH-SY5Y) cell line was kindly provided by Dr. Panicker, National Centre for Biological Sciences, Bangalore, human glioblastoma (U-87 MG) cells by Dr. Nandakumar, NIMHANS, human microglial (CHME-3) cells by Dr. Anirban Basu, National Brain Research Center, Gurgaon and rat glioma (C6) cell line was provided by Dr. Kumar, IISc, Bangalore. SH-SY5Y cells were grown and maintained in Dulbecco Modified Essential Medium (DMEM)/F12 (Gibco, USA) supplemented with 10% heat-inactivated FBS, 100 U/ml penicillin, 100 μg/ml streptomycin (Life Technologies) in humidified 5% CO_2_ at 37 °C. U-87 MG, CHME-3, C6 and Rhesus monkey kidney (LLC-MK2) cells were cultured in DMEM containing 10% FBS at 37 °C and 5% CO_2_. DENV-3 was titrated by standard plaque assay on LLC-MK2. All the cells were tested for mycoplasma contamination and found to be negative.

### Antibodies

Dengue-3 serotype-specific monoclonal antibody (D6-8A1–12) and flavivirus group-specific monoclonal antibody (4G2) were kindly provided by Dr. Barbara Johnson, CDC, Fort Collins, USA. Goat anti-mouse IgG Horseradish peroxidase (HRP) conjugate and Goat anti-rabbit IgG HRP conjugate (Genie, India), anti-prohibitin polyclonal antibody (pAb), anti-prohibitin-2 (pAb) and anti-vimentin (pAb) antibodies were procured commercially (Sigma Aldrich, USA). The Cy3 labelled anti-rabbit antibody was procured from Thermo Scientific, USA. The recombinant DENV-3 EDIII protein was procured from ProSpec-Tany TechnoGene Ltd., Israel.

### Growth and purification of DENV-3 obtained from infected tissue culture fluid

The DENV-3 infectious cell culture fluid was concentrated as described earlier [15] with minor modifications. Briefly, virus infected C6/36 supernatant fluid was collected at 5 days post infection (PI) and clarified by centrifugation at 1000 X g for 10 min. Virus particles were precipitated from the supernatant using polyethylene glycol (PEG, MW 8000; Sigma, USA) using 7% PEG and 2.4% NaCl (w/v at the final concentration) while stirring on ice for 20 min. The mixture was kept at 4 °C overnight and centrifuged at 14000 X g at 4 °C for 60 min to obtain the virus- rich precipitate. The virus pellet was re-suspended in TNE buffer (10 mM Tris-HCl, 100 mM NaCl, 1 mM EDTA, pH 7.8) in 1/100th of the original volume. The DENV-3 virus was further purified by overlaying concentrated virus suspension onto a discontinuous sucrose gradient of 30–60% (w/v) in TNE buffer and ultra centrifuged at 80,000 X g (Beckman SW 41Ti rotor) at 4 °C for 18 h. Fractions were collected from the gradient, re-suspended in TNE buffer and stored at − 70 °C. The virus infectivity was tested by plaque assays in LLC-MK2 cells. A single stock of DENV-3 was used for all experiments.

### Membrane protein preparation

Cell membrane proteins of SH-SY5Y, U-87 MG and CHME-3 were prepared as described previously [16]. Briefly, six T-150 culture flasks of confluent cells were washed three times with Tris-buffered saline [TBS- 50 mM Tris HCl (pH 7.6), 150 mM NaCl]. Cells were detached by scrapping and pellet was collected by centrifugation at 600 X g for 5 min. Supernatant was discarded and cells were re-suspended in ice-cold Buffer M [20 mM Tris-HCl (pH 8), 100 mM NaCl, 2 mM MgCl_2_, 1 mM EDTA, 0.2% Triton X-100], homogenised by vortexing and incubated for 20 min on ice. Further, cells were centrifuged at 610 X g for 3 min to remove nuclei and cell debris. This step was repeated thrice to ensure complete lysis. Supernatants were pooled and centrifuged at 6000 X g for 5 min to remove membrane organelles. To obtain membrane protein, the supernatant was further pelleted by centrifugation at 20,800 X g for 20 min. Resulting pellet was dissolved in Buffer M containing 1X protease inhibitor and stored at − 70 °C. The concentration of the cell membrane protein was determined by Nanodrop (Thermo Scientific, USA). The purity of cell membrane protein preparation was determined by Western blot using voltage-dependent anion channel (VDAC) antibody (Abcam), a specific membrane marker [17].

### Virus overlay protein binding assay (VOPBA)

To determine DENV-3 binding to molecules present on the plasma membrane of SH-SY5Y, U-87 MG, CHME-3 cells and C6 cells (non-susceptible to DENV), VOPBA was performed as described earlier [16] with minor modifications. Cell membrane proteins (100 μg/well) were resolved by 12% SDS-PAGE and transferred to nitrocellulose membrane using iBlot 2 gel transfer device (dry blotting). Following overnight blocking with 5% skimmed milk in phosphate-buffered saline (PBS) containing 0.05% Tween 20 (PBST), the membrane was washed with PBST thrice for 5 min followed by incubation with 10^6^ pfu of purified DENV-3 for 3 h at 37 °C. The membrane was washed with PBST thrice for 5 min each. Binding of dengue virus to the membrane was detected by further incubation with flavivirus group-specific monoclonal antibody at a dilution of 1:400 overnight at 4 °C, followed by washing five times with PBST. The membrane was subsequently incubated with secondary horseradish peroxidase-conjugated rabbit anti-mouse IgG (GeneTex, USA) followed by five washes with PBST. Cell membrane protein/(s) interacting with DENV serotypes was visualized by chemiluminescence using a commercial substrate (Super Signal West Pico, Thermo Scientific, USA).

### Liquid chromatography-mass spectrometry (LC-MS/MS) analysis

The nitrocellulose blot post-VOPBA was aligned with SDS-PAGE gel stained with Coomassie Brilliant Blue. With the help of a sterile scalpel the corresponding positive band/s was excised, immersed in Ammonium Bicarbonate (ABC) Buffer and subjected to in-gel digestion following a protocol described earlier [18]. The excised gel pieces were transferred to sterile microfuge tubes and washed with a de-staining solution (40% Acetonitrile in 40 mM ammonium bicarbonate) by shaking vigorously. After the gel pieces were completely de-stained, the supernatant was discarded by a brief centrifugation at 3000 rpm. Gel pieces were dehydrated using 100% acetonitrile (ACN) for 2–3 min until it shrunk and turned opaque. Proteins present in gel pieces were further reduced by 10 mM Dithiothreitol (DTT) in 40 mM ABC buffer and incubated at 60 °C for 30–40 min and then alkylated using 10 mM iodoacetamide in 40 mM ABC and further incubated at room temperature for 20 min followed by dehydration by 100% ACN. The proteins were digested by adding sequencing grade trypsin (Promega, USA), enough to cover the gel pieces at a concentration of 10 ng/μl in 40 mM ABC buffer on ice for 10 min followed by overnight incubation at 37 °C. The supernatants were transferred to fresh microfuge tubes and the digested peptides were extracted twice by 100 μl of extraction buffer (40% ACN in 5% formic acid) for 10 min on a shaker. The final extraction was done with 100% ACN buffer, dried by speed-vac at 45 °C and kept at − 70 °C until LC-MS/MS analysis was carried out.

The peptides were analyzed using LTQ-Orbitrap Fusion mass spectrometer (Thermo Scientific, Germany) interfaced with Easy-nLC 1000 (Thermo Scientific, Germany). The dried peptides were reconstituted in 40 μl of 0.1% formic acid and loaded onto a trap column (2 cm, 5 μ–100A^o^) packed with magic AQ C18 material (Michrom Bioresources, USA) and resolved using an analytical column (15 cm, 3 μ–100A^o^). The solvent system included solvent A (0.1% formic acid) and solvent B (95% ACN in 0.1% formic acid). A gradient of 7% solvent B to 35% solvent B was used over 30 min for the analysis. A mass resolution of 120,000 was used for MS scans with AGC target of 200,000 and ion injection time of 55 milliseconds. Peptides with 2–6 charge were selected for further fragmentation and dynamic exclusion of 30 s was enabled. Most abundant precursor ions were fragmented using HCD fragmentation (34% collision energy) and fragment ions were acquired in a range of 100–2000 m/z with Orbitrap mass resolution of 30,000.

The raw files were searched using SEQUEST search engine against the Human RefSeq protein (NCBI) through Proteome Discoverer suite, version 2 (Thermo Scientific, Germany). Trypsin was selected as the enzyme with single missed cleavage and a precursor mass tolerance of 20 ppm and fragment ion mass tolerance of 0.05 Da was used for the searches. Percolator node was used to compute False Discovery Rate (FDR) and an FDR of 1% at Peptide Spectrum Matches (PSM) level was applied.

### Infection inhibition assay to establish role of cell surface proteins

To ascertain the role of prohibitin1 (PHB1)/ prohibitin2 (PHB2) and vimentin (VIM) in entry of DENV-3 into SH-SY5Y cells and the role of PHB1/2 in entry of DENV-3 into U-87 MG and CHME-3 cells, an infection inhibition assay was performed using polyclonal antibody against PHB1, PHB2 and VIM in a dose-dependent manner. Monolayer of SHSY5Y/ U-87 MG/ CHME-3cells (0.04 X 10^6^ cells/ml) grown on coverslips in 24 well plates and pre-incubated with anti PHB1 antibody (1 μg and 2 μg), anti PHB2 antibody (1 μg and 2 μg) or anti-VIM antibody (1:20 and 1:40) in medium for 4 h on a rocking platform. Anti-actin antibody (1: 100) (Sigma-Aldrich, USA) was used as non-specific antibody control. Untreated cells were used as virus control. The cells were washed and subsequently infected with DENV-3 at 10 MOI at 37 °C for 2 h. Excess or unbound virus was removed by washing and cells were replenished with fresh medium. After 30 h PI, cells were fixed in chilled methanol and presence of DENV-3 antigen was determined by indirect immunofluorescence assay (IFA). Briefly, coverslips were washed twice with sterile PBS, permeabilized using 0.3% Triton X-100 for 10 min at room temperature and blocked with 5% BSA at 37 °C for 30 min. Cells were incubated with serotype- specific DENV-3 monoclonal antibody at 37 °C for 2 h, subjected to three washes using PBST and subsequently stained with anti-mouse FITC conjugated secondary antibody (Life Technologies, USA) for 1 h at 37 °C. After washing thrice with PBST, the coverslips were mounted on clean glass slides and observed under a fluorescence microscope (Eclipse TS 100; Nikon, Japan). Uninfected cells were included as a control. Further, the cell culture supernatant collected at 30 h PI was used to enumerate the extracellular viral particles by plaque assay as described earlier [16, 19]. All the experiments were performed in triplicates. For semiquantitative evaluation of fluorescence by microscopy, five representative fields in various parts of the smear were examined. In each of these representative fields, using a grid, the number of cells fluorescing were first noted and without altering the field the UV filter was shifted to phase contrast and the total number of cells in the field was counted. The percentages of fluorescent positive cells in each of the five fields were enumerated and an average was derived.

### Surface localization of DENV-3-interacting protein/s on SH-SY5Y, U-87 MG, and CHME-3 cells

To reconfirm the distribution of membrane and cytoplasmic PHB1/2, IFA was performed on uninfected SH-SY5Y/ U-87 MG/ CHME-3 cells. Cells (0.4 X 10^6^) were seeded on coverslips in a 24-well tissue culture plate and incubated at 37 °C. After 24 h, cells were fixed with 2% paraformaldehyde at room temperature. One set of cells were permeabilized using 0.5% Triton X-100. IFA was performed using anti-PHB1/2 antibody followed by FITC as mentioned earlier. The anti-DENV-3 monoclonal antibody was used as negative control.

### Co-immunoprecipitation and Western blot analysis

Co-immunoprecipitation (Co-IP) assay was performed as described earlier [19] with modifications. Briefly, SH-SY5Y/ CHME-3 cell membrane protein (150 μg) was incubated with DENV-3 EDIII protein or without virus (control). The proteins were incubated in 2X immuno-precipitation (IP) buffer (2% Triton X-100 and 0.1% NP 40) with gentle rocking at 4 °C for 2 h. Subsequently, the complex was incubated with anti-PHB1/2 antibody overnight at 4 °C on a rocker. This immune-complex was then pulled down by 50% slurry of Protein G sepharose beads (Protein G PLUS-Agarose, Santa Cruz, USA) with gentle rocking at 4 °C for 4 h. After centrifugation, the pellet was washed thrice with 1X IP buffer and finally re- suspended in SDS sample buffer. The protein complex was heated at 100 °C for 5 min followed by centrifugation at 14,000 X g for 5 min at 4 °C. The supernatant was collected and proteins were resolved by 12% SDS-PAGE followed by Western blotting on nitrocellulose membrane. The presence of DENV-3 EDIII protein in the DENV-3 complexes- (PHB1/2) was detected using anti-flavivirus group-specific monoclonal antibody (4G2) followed by incubation with IgG horseradish peroxidase-conjugated secondary antibody. The band was developed by chemiluminescence. Additionally, the SH-SY5Y/ CHME-3 membrane protein- DENV-3 immune complex was incubated with anti-DENV antibodies and subsequently separated by Protein G sepharose beads. After washing with 1X IP buffer, proteins were resolved by 12% SDS-PAGE followed by Western blot. The presence of PHB1/2 proteins in the complex was detected by anti-PHB1/2 antibodies.

### Double labelling

SH-SY5Y and CHME-3 cells were grown on coverslips in 24 well tissue culture plates until 60% confluence. Subsequently, the cells were infected with DENV-3 at MOI of 10. At 24 h PI, the cell culture media was removed and cells were washed with 1XPBS and fixed with 100% ice-cold methanol for 20 min. Fixed SH-SY5Y /CHME-3 cells were washed with PBS, blocked with 5% BSA for 30 min at 37 °C. Cells were incubated with DENV-3 anti-envelope protein monoclonal antibody at 37 °C for 2 h followed by incubation with anti-mouse FITC conjugated secondary antibody for 1 h at 37 °C. One set of these stained cells were further incubated with anti-PHB1 rabbit polyclonal antibody while the other set was incubated with anti-PHB2 antibody for double labelling at 37 °C for 2 h. The cells were subsequently washed and incubated with secondary goat anti-rabbit antibody conjugated to Cy3 for 2 h at room temperature in a humid chamber. Coverslips were washed with PBST (5 times) and mounted on glass slides and observed under a confocal microscope (Leica TCS, Germany). Appropriate controls were included in the assay.

### Flow cytometric analysis for surface expression of membrane-interacting proteins

SH-SY5Y cells were grown in T25 flasks to 80% confluence. Cells were then infected with DENV-3 (MOI 10). Uninfected cells were used as a control. Cells were harvested at 0 h, 24 h, 48 h and 72 h PI by scrapping, washed with PBS and incubated with 1:50 dilution of anti-PHB1/2 antibody at 4 °C for 40 min. Cells were subsequently stained with FITC conjugated secondary antibody and analyzed by flow cytometry for surface expression of PHB1/2 (FACS Verse, BD Biosciences, USA) using Flowjo software (TreestarInc, USA). U87-MG cells were taken as negative control for PHB1 surface expression.

### Small interfering RNA (siRNA) transfection

siRNA duplexes targeting genes encoding for human PHB1 (SR303488), PHB2 (SR307765) and scrambled negative control siRNA duplex (SR30004) were purchased from OriGene. SH-SY5Y, CHME-3 and U-87 MG cells (5 × 10^4^ cells / well) were reverse transfected with 100 nM of each of the siRNAs using Lipofectamine RNAiMAX reagent (Invitrogen) in 24 wells plate. At 48 h post transfection, cells were infected with DENV-3 at MOI 10. Culture supernatant and cell lysate were harvested at 30 h PI for viral quantification by plaque assay and Western blot analysis.

### Western blot analysis

Cells were washed with ice cold PBS and lysed in radioimmunoprecipitation assay(RIPA) buffer for 30 min on ice (50 mM tris HCl, 150 mM NaCl, 0.1% triton-100, 0.5% sodium deoxycholate, 0.1% SDS and 1 mM NaF Protease inhibitor). Supernatant were collected after centrifugation. Further, 20 μg of total protein were resolved in 12% SDS-PAGE and transferred onto nitrocellulose membrane using semidry transfer system (Biorad). After blocking with 5% skimmed milk in PBST for 2 h at RT, the membranes were incubated with PHB1/ PHB2/ β-actin antibodies (Sigma Aldrich, USA) overnight. After washing five times with PBST, the membranes were subsequently incubated with secondary horseradish peroxidase-conjugated goat anti-rabbit IgG (GeneTex, USA) followed by five washes with PBST. The chemiluminescence signal was visualized by ECL substrate (Super Signal West Pico, Thermo Scientific, USA).

### Virus attachment and entry assays

CHME-3 and U-87 MG cells were seeded in 96 well plate (1 × 10^4^ cells/ well). Cell monolayer was pre-chilled at 4 °C for 1 h. The monolayer was co-treated with DENV-3 (MOI 100) and PHB1/2 polyclonal antibodies at 4 °C for 1.5 h (attachment assay) as described [20]. Unbound virus was removed by washing with ice-cold PBS three times. Cells were replenished with fresh medium and incubated at 37 °C for 30 h. The entry assay was carried out by incubation of DENV-3 (MOI 100) with cells at 4 °C for 1.5 h to allow binding, and unbound virus was removed by washing with ice-cold PBS. Cells were then treated with PHB1/2 polyclonal antibodies at 37 °C for 1.5 h to allow internalization. Non-internalized virions were washed with PBS (thrice) and cells were replenished with fresh medium and incubated at 37 °C for 30 h. Supernatant was harvested 30 h PI from both assays and virus was quantified by plaque assay. Each experiment was performed in triplicates.

### Statistical analysis

Data from infection inhibition were analyzed using the GraphPad Prism 5 (GraphPad Software Inc., Calif.). One way ANOVA followed by post-test by Bonferroni correction was employed. *p* < 0.05 was considered significant.

## Results

### Purity of cell membrane preparation

**Fig. 1:**
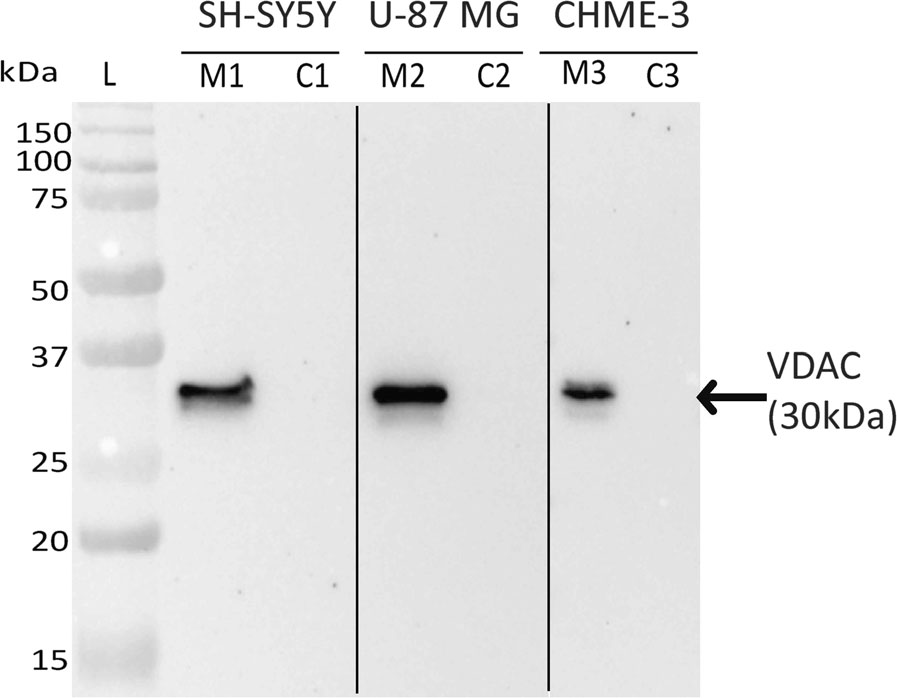
Purity of membrane and cytosolic fractions isolated from SH-SY5Y, U-87 MG and CHME-3 cells. The purity of protein fractions were assessed by western blotting against VDAC membrane marker. Note the presence of VDAC in membrane fractions (M1, M2 and M3) and absence of VDAC in cytosolic fractions (C1, C2 and C3) of SH-SY5Y, U-87 MG and CHME-3 cells

The purity of cell membrane preparation of SH-SY5Y, U-87 MG and CHME-3 were assessed by western blotting against membrane specific marker using VDAC antibody (Abcam). A 30 kDa band (VDAC) was observed in the membrane fractions of all the cells but absent in the cytosolic fractions. These results confirmed the purity of the membrane fractions prepared from the cells (Fig. 1).

### Identification of surface molecules on neural cells interacting with DENV-3

**Fig. 2:**
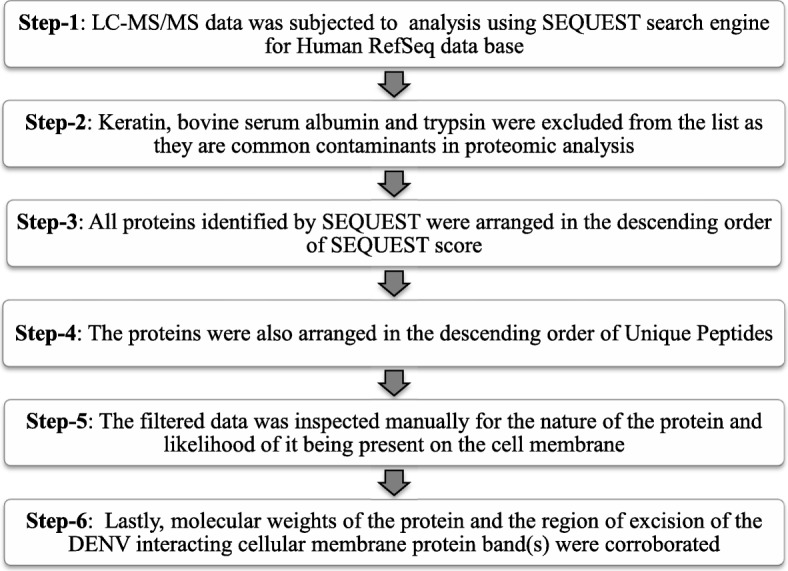
The algorithm adopted to identify DENV interacting protein(s) on the surface of cells

As a large number of interacting proteins were obtained by SEQUEST search, an algorithmic approach described earlier [21] with minor modification was adopted to identify the most likely interacting proteins. The algorithm used SEQUEST scores, unique peptide, and molecular mass data followed by the probability of the interacting protein being present on the surface (Fig. 2). The list of proteins obtained following algorithmic analysis is presented in Tables 1, 2, and 3.

**Table 1:** SEQUEST search results LC-MS/MS analysis of DENV-3 binding protein on SH-SY5Y cell membrane

Accession	Gene symbol	Description	Sequest Score	PSMs	Unique Peptides	MW [kDa]
A						
**62414289**	**VIM**	**vimentin**	**1.85**	**1**	**1**	**53.62**
32189394	ATP5B	ATP synthase subunit beta, mitochondrial precursor	2.78	1	1	56.53
4501881	ACTA1	actin, alpha skeletal muscle	1.64	1	1	42.02
B						
**221307584**	**PHB2**	**prohibitin-2 isoform 1**	**25.53**	**9**	**8**	**33.276**
5174447	GNB2L1	guanine nucleotide-binding protein subunit beta-2-like 1	21.85	8	5	35.055
4506663	RPL8	60S ribosomal protein L8	18.08	5	4	28.007
4507879	VDAC1	voltage-dependent anion-selective channel protein 1	17.83	7	4	30.754
17158044	RPS6	40S ribosomal protein S6	15.62	5	5	28.663
386869503	RPS3	40S ribosomal protein S3 isoform 2	14.90	7	3	28.468
4506661	RPL7A	60S ribosomal protein L7a	14.40	5	5	29.977
208879465	VDAC3	voltage-dependent anion-selective channel protein 3 isoform 2	8.02	3	1	30.77
4502709	CDK1	cyclin-dependent kinase 1 isoform 1	7.73	3	3	34.074
15431301	RPL7	60S ribosomal protein L7	6.28	2	2	29.207
C						
156071459	SLC25A5	ADP/ATP translocase 2	282.03	107	15	32.83
4506725	RPS4X	40S ribosomal protein S4, X isoform X isoform	210.99	89	28	29.58
55749577	SLC25A4	ADP/ATP translocase 1	158.48	63	4	33.04
**528281407**	**PHB**	**prohibitin isoform 1**	**85.93**	**33**	**15**	**29.79**
208973244	YWHAZ	14–3-3 protein zeta/delta	77.24	32	14	27.73
4505753	PGAM1	phosphoglycerate mutase 1 isoform 1	61.48	20	15	28.79
316659409	ACTG1	actin, cytoplasmic 2	55.26	24	10	41.77
4507949	YWHAB	14–3-3 protein beta/alpha	46.59	19	5	28.07
5803227	YWHAQ	14–3-3 protein theta	43.68	20	9	27.75
21464101	YWHAG	14–3-3 protein gamma	38.78	17	8	28.29

Proteins are arranged in descending order based on scores obtained (A) ~ 50 kDa protein (B) ~ 31 kDa protein (C) ~ 28 kDa. VIM, 53 kDa proteins, PHB2, 33 kDa protein and PHB2, 30 kDa proteins were identified as bona fide protein to serve as a DENV-3 receptor on SH-SY5Y cells. Bold fonts represent the proteins identified in this study for further characterization based on the results obtained in the algorithm

**Table 2:** SEQUEST search results of LC-MS/MS analysis of DENV-3 binding protein on U-87 MG cell membrane

Accession	Gene symbol	Description	Sequest Score	PSMs	Unique Peptides	MW [kDa]
A						
4507879	VDAC1	voltage-dependent anion-selective channel protein 1	203.68	106	16	30.75
**221307584**	**PHB2**	**prohibitin-2 isoform 1**	**113.01**	**42**	**15**	**33.28**
4507651	TPM4	tropomyosin alpha-4 chain isoform Tpm4.2cy	86.45	32	12	28.50
296317337	VDAC2	voltage-dependent anion-selective channel protein 2 isoform 1	74.70	37	10	33.35
4502317	ATP6V1E1	V-type proton ATPase subunit E 1 isoform a	32.81	14	11	26.13
23238211	ARPC2	actin-related protein 2/3 complex subunit 2	31.23	12	10	34.31
94721252	VAPA	vesicle-associated membrane protein-associated protein A isoform 2	29.45	11	7	27.88
4503477	EEF1B2	elongation factor 1-beta	27.62	12	5	24.75
B						
156071459	SLC25A5	ADP/ATP translocase 2	147.37	66	10	32.83
**528281407**	**PHB**	**prohibitin isoform 1**	**125.86**	**53**	**14**	**29.79**
208973244	YWHAZ	14–3-3 protein zeta/delta	124.19	72	14	27.73
156071462	SLC25A6	ADP/ATP translocase 3	111.96	51	6	32.85
5803227	YWHAQ	14–3-3 protein theta	109.82	49	13	27.75
4507949	YWHAB	14–3-3 protein beta/alpha	91.79	39	6	28.07
4505753	PGAM1	phosphoglycerate mutase 1 isoform 1	91.30	38	14	28.79
21464101	YWHAG	14–3-3 protein gamma	59.11	26	6	28.29
4503727	FKBP3	peptidyl-prolyl cis-trans isomerase FKBP3	58.55	24	14	25.16
15431297	RPL13	60S ribosomal protein L13 isoform 1	49.72	21	13	24.25

Proteins are arranged in descending order based on scores obtained (A) ~ 31 kDa protein (B) ~ 28 kDa protein. PHB2, 33 kDa protein and PHB1 30 kDa protein was identified as a bona fide protein to serve as a DENV-3 receptor on U-87 MG cells. Bold fonts represent the proteins identified in this study for further characterization based on the results obtained in the algorithm

**Table 3:** SEQUEST search results of LC-MS/MS analysis of DENV-3 binding protein on CHME-3 cell membrane

Accession	Gene symbol	Description	Sequest Score	PSMs	Unique Peptides	MW [kDa]
A						
5174447	GNB2L1	guanine nucleotide-binding protein subunit beta-2-like 1	118.74	59	15	35.06
4506661	RPL7A	60S ribosomal protein L7a	70.36	26	18	29.98
5803225	YWHAE	14–3-3 protein epsilon	54.73	26	13	29.16
4507879	VDAC1	voltage-dependent anion-selective channel protein 1	43.06	18	9	30.75
94721250	VAPA	vesicle-associated membrane protein-associated protein A isoform 1	29.30	10	6	32.59
50345988	ATP5C1	ATP synthase subunit gamma, mitochondrial isoform L (liver) precursor	28.24	10	8	32.98
296317337	VDAC2	voltage-dependent anion-selective channel protein 2 isoform 1	26.15	10	7	33.35
4502901	CLTB	clathrin light chain B isoform a	23.83	10	10	23.17
4502709	CDK1	cyclin-dependent kinase 1 isoform 1	20.89	10	7	34.07
4502107	ANXA5	annexin A5	18.92	9	8	35.91
4505581	PRKRA	interferon-inducible double-stranded RNA-dependent protein kinase activator A isoform 1	18.66	8	5	34.38
4502317	ATP6V1E1	V-type proton ATPase subunit E 1 isoform a	15.38	8	5	26.13
**221307584**	**PHB2**	**prohibitin-2 isoform 1**	**9.82**	**4**	**4**	**33.28**
20149675	EFHD2	EF-hand domain-containing protein D2	8.92	4	3	26.68
4759302	VAPB	vesicle-associated membrane protein-associated protein B/C isoform 1	8.49	4	3	27.21
16936528	CDK2	cyclin-dependent kinase 2 isoform 1	8.35	5	4	33.91
208879465	VDAC3	voltage-dependent anion-selective channel protein 3 isoform 2	7.32	4	3	30.77
156071459	SLC25A5	ADP/ATP translocase 2	7.20	3	3	32.83
B						
156071459	SLC25A5	ADP/ATP translocase 2	79.76	37	8	32.83
156071462	SLC25A6	ADP/ATP translocase 3	72.55	35	3	32.85
**528281407**	**PHB**	**prohibitin isoform 1**	**68.55**	**35**	**13**	**29.79**
4506727	RPS4Y1	40S ribosomal protein S4, Y isoform 1	43.40	18	4	29.44
5803227	YWHAQ	14–3-3 protein theta	42.59	22	9	27.75
4759302	VAPB	vesicle-associated membrane protein-associated protein B/C isoform 1	39.07	16	12	27.21

Proteins are arranged in descending order based on scores obtained (A) ~ 31 kDa protein (B) ~ 28 kDa protein. PHB2, 33 kDa protein and PHB1 30 kDa protein was identified as a bona fide protein to serve as a DENV-3 receptor on CHME-3 cells. Bold fonts represent the proteins identified in this study for further characterization based on the results obtained in the algorithm

**Fig. 3:**
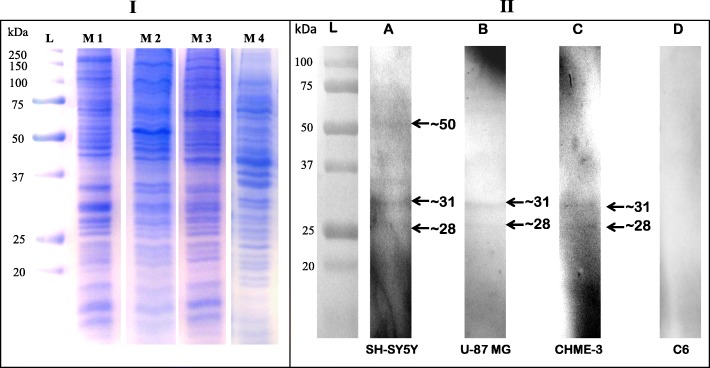
Virus overlay protein binding assay (VOPBA) with SH-SY5Y, U-87 MG, CHME-3 and C6 cell membrane proteins. Membrane proteins from neural cells were resolved by SDS-PAGE and transferred to nitrocellulose membrane. DENV-3 was overlaid on to the nitrocellulose membrane. The interacting proteins were detected by using flavivirus group-specific monoclonal antibody (4G2). **Panel-I:** Coomassie Brilliant Blue stained gel image depicting various cell membrane proteins. Membrane proteins fractions from SH-SY5Y (M1), U-87 MG (M2), CHME-3 (M3) and C6 (M4) were resolved by 12% SDS-PAGE. Lane L indicates the protein molecular weight ladder. **Panel-II:** VOPBA of neural cell membrane proteins using DENV-3. Lane A, B, C and D depicts overlay on cell membrane protein of SH-SY5Y, U-87 MG, and CHME-3 respectively. Note the presence of ~ 31 and ~ 28 kDa bands (arrow) across the three neural cell lines exhibiting positive interaction and no band in C6 cell line (negative control)

To identify proteins present on the neural cell surface that interact with DENV-3 serotype, VOPBA was carried out followed by LC-MS/MS analysis. As evident from the Fig. 3, Panel II, three bands of molecular mass ~ 50, ~ 31 and ~ 28 kDa interacting with DENV-3 were identified on SH-SY5Y cell membrane (lane A), whereas, two bands of molecular mass ~ 31 kDa and ~ 28 kDa were identified on U-87 MG (lane B) as well as CHME-3 cell membranes (lane C). No band was observed on C6 cell membrane (lane D).

### LC-MS/MS mass spectrometry followed by SEQUEST search

Based on the results obtained in the algorithm, VIM (MW 53.62 kDa), PHB1 (MW 30 kDa) and PHB2 (MW 33.28 kDa) were identified as interacting proteins on the SH-SY5Y cell surface (Table 1). Similarly, PHB1 (MW 30 kDa) and PHB2 (MW 33.28 kDa) were identified as interacting proteins on U-87 MG and CHME-3 cells (Tables 2, 3). The other proteins were not considered for further analysis as they did not fulfil the requirements of the algorithm.

### Infection inhibition assay to characterize identified molecules in DENV-3 entry in neural cells

**Fig. 4:**
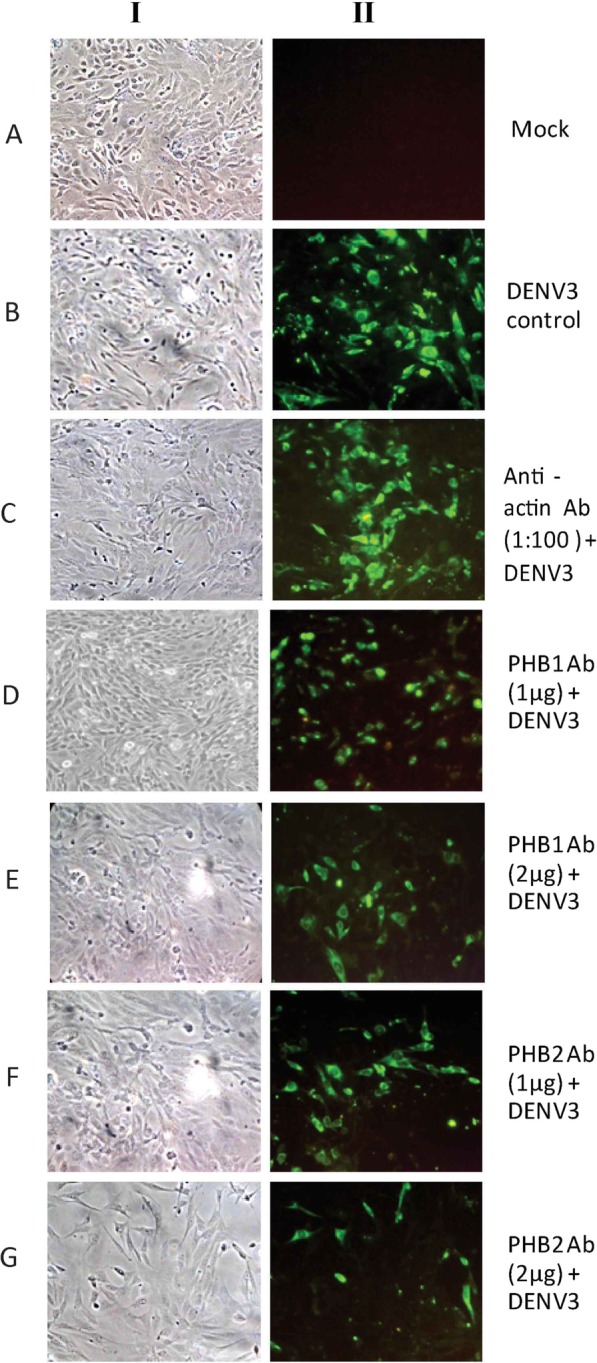
A representative image depicting infection inhibition assay using anti-PHB1/2 antibody-IFA. SH-SY5Y cells were infected with DENV-3 in the absence and presence of anti-PHB1/2. **Panel I:** Phase contrast microscopic images. **Panel II:** Detection of DENV-3 antigen by IFA 30 h PI. Untreated cells and cells treated with anti-actin antibody served as a control. Note the reduction in fluorescence in the cells incubated with anti-PHB1/2 antibodies as compared to controls (Panel B)

The VOPBA followed by LC-MS/MS analysis suggested that PHB1/2 and VIM could be involved in internalization of DENV-3 into SH-SY5Y cells while PHB1/2 could be involved in entry into U-87 MG and CHME-3 cells. Pre-incubation of SH-SY5Y cells with the anti-PHB1/2 antibody (Fig. 4) and CHME-3 cells with anti-PHB1/2 exhibited a reduction in fluorescence when stained for DENV-3 antigen in a dose-dependent manner. However, pre-incubation of SH-SY5Y cells with the anti-VIM antibody and U-87 MG cells with PHB1/2 antibodies did not reveal any difference in DENV-3 antigen expression between control and antibody treated cells. Further, the fluorescence noted with DENV-3 infected cells pre-incubated with anti-actin antibody was same as virus control suggesting that the observations noted with anti-PHB1/2 antibodies were indeed specific (Fig. 4). The percentage of cells fluorescing reduced from 40% (virus control) to 17 and 10% with cells treated with two different concentrations of anti PHB1 antibody and to 11 and 6% with cells treated with two different concentrations of anti PHB2 antibody (Table 4).

**Table 4:** Semi quantitative analysis of IFA results of infection inhibition assay

	Total No. of cells ^a^	No. of cells fluorescing^a^	% fluorescent positive cells
DENV3 virus control	325	129	40
Actin Ab (1:100) + DENV3	346	120	35
PHB1 Ab (1 μg) + DENV3	300	52	17
PHB1 Ab (2 μg) + DENV3	348	35	10
PHB2 Ab (1 μg) + DENV3	333	39	11
PHB2 Ab (2 μg) + DENV3	313	6	6

^a^Average number of cells calculated from five different fields

**Fig. 5:**
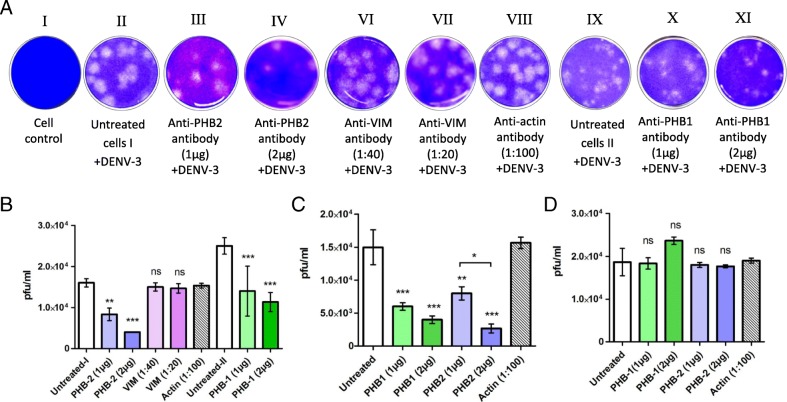
Antibody-mediated inhibition of DENV-3 infection in SH-SY5Y, CHME-3 and U-87 MG cells. Cells were pre-treated with anti-PHB1/2 or anti-VIM antibody at two different concentrations. **A** Representative picture depicting plaques obtained in antibody pre treated and untreated SH-SY5Y cells. The virus yield (pfu/ml) obtained in infected **B** SH-SY5Y cells **C** CHME-3 cells and **D** U-87 MG cells in triplicate experiments are displayed by error bars (mean ± SD). Statistics are compared to untreated controls. Note the significant dose-dependent inhibition of DENV-3 infection in SH-SY5Y and CHME-3 cells treated with anti-PHB-1/2 (*p* < 0.0001) while, there was no reduction in titers obtained in cells treated with anti-PHB1/2 antibody as compared with uninfected U-87 MG cells. No significant inhibition was observed with cells treated with anti-VIM. (ns *p* > 0.5, *p < 0.01, ** *p* < 0.001, *** *p* < 0.0001)

A plaque reduction assay was performed to further explore the role of PHB1/2 and VIM in DENV-3 entry into SH-SY5Y cells, PHB1/2 for entry into U-87 MG and CHME-3 cells. A significant reduction in DENV-3 plaques was noted in SH-SY5Y cells pre-incubated with anti-PHB1 (55%) and anti-PHB2 (75%) antibodies (Fig. 5). Similarly, a reduction of 82.2 and 73.3% in DENV-3 plaques were observed upon pre-incubation of CHME-3 cells with PHB2 and PHB1 antibody respectively (Fig. 5). On the contrary, no inhibition of infection was noted in SH-SY5Y cells when treated with anti-VIM antibody or in U-87 MG cells when treated with anti-PHB1/2 antibodies (Fig. 5).

### Cell surface localization of PHB1/2 on SH-SY5Y/ U-87 MG/ CHME-3 cells

**Fig. 6:**
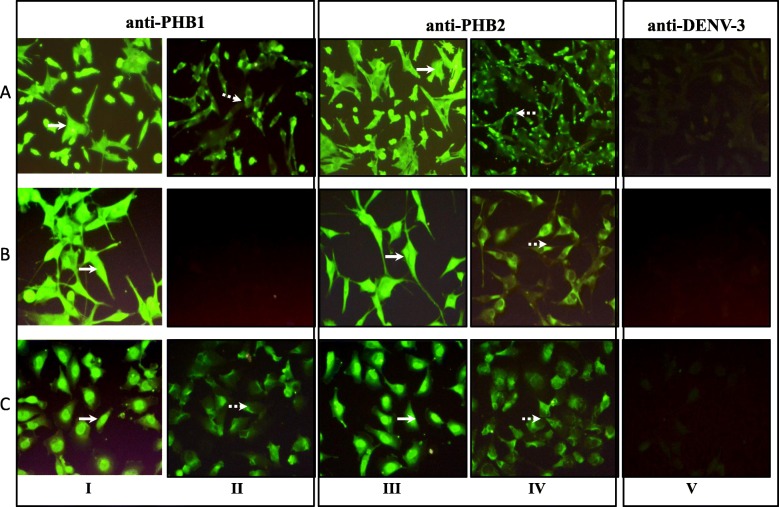
Localization of PHB1/2 on the surface of SH-SY5Y, U-87 MG and CHME-3 cells. Permeabilized and non-permeabilized SH-SY5Y (Panel A), U-87 MG (Panel B), and CHME-3 (Panel C) cells were stained with PHB1 **(Panel I and II)** and PHB2 **(Panel III and IV)** antibody separately while anti-DENV-3 antibody **(Panel V)** was used as negative antibody control. Panel I, III and V represents permeabilized cells and Panel II and IV represents the non-permeabilized cells. Note that both permeabilized (solid white arrow) and non-permeabilized cells (dotted white arrow) stained positive for PHB1 (Panel A) and PHB2 (Panel C) on SH-SY5Y and CHME-3 cells while non-permeabilized U-87 MG cells did not exhibit fluorescence for PHB1

Presence of PHB1/2 on the surface of permeabilized and non-permeabilized SH-SY5Y/ U-87 MG/ CHME-3 cells was confirmed by IFA (Fig. 6). The presence of PHB2 was noted in the cytoplasm (permeabilized) as well as on the cell surface (non-permeabilized) of SH-SY5Y, U-87 MG and CHME-3 cells. On the other hand, PHB1 was not detected on the surface of U-87 MG cells (non-permeabilized).

### Co-immunoprecipitation assay and double labelling confirms the interaction of PHB1/2 with DENV-3 E proteins

**Fig. 7:**
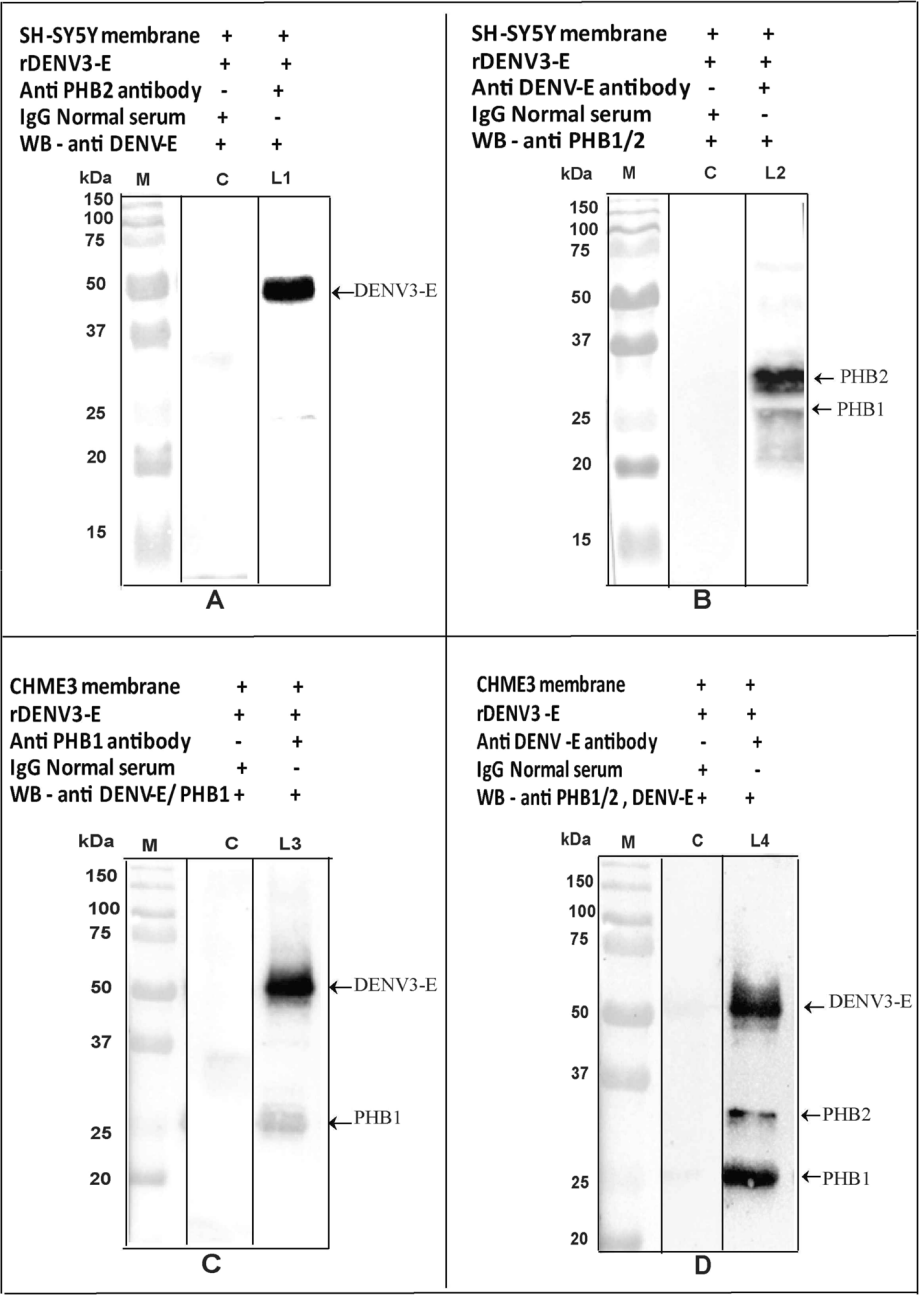
Western Blot of Co-IP demonstrates the interaction between PHB1/2 and DENV-3 E proteins on SH-SY5Y/ CHME-3 cell membrane. **A** The presence of DENV-3 E protein (45 kDa) in the complex (SH-SY5Y membrane + DENV-3 incubated with anti PHB2 antibody was confirmed (arrow) by Western blot analysis using anti-DENV-3 antibody (L1). **B** The presence of PHB1/2 protein in the complex (SH-SY5Y membrane + DENV-3 incubated with anti DENV antibody was confirmed (arrow) by Western blot analysis using anti-PHB1/2 antibody (L2). **C** The presence of DENV-3 E protein and PHB1 (30 kDa) protein in the complex (CHME-3 membrane + DENV-3 incubated with anti-DENV antibody) was confirmed (arrow) by Western blot analysis using anti-PHB1 and anti-DENV-3 antibodies (L3). **D** The presence of PHB1/2 protein and DENV-3 E protein in the complex (CHME-3 membrane + DENV-3 incubated with anti-DENV antibody) was confirmed (arrow) by Western blot analysis using anti-PHB1/2 and anti-DENV antibody (L4). Lane M represents the molecular weight of protein marker. Lane C represents the absence of DENV-3 E protein, run as a control

**Fig. 8:**
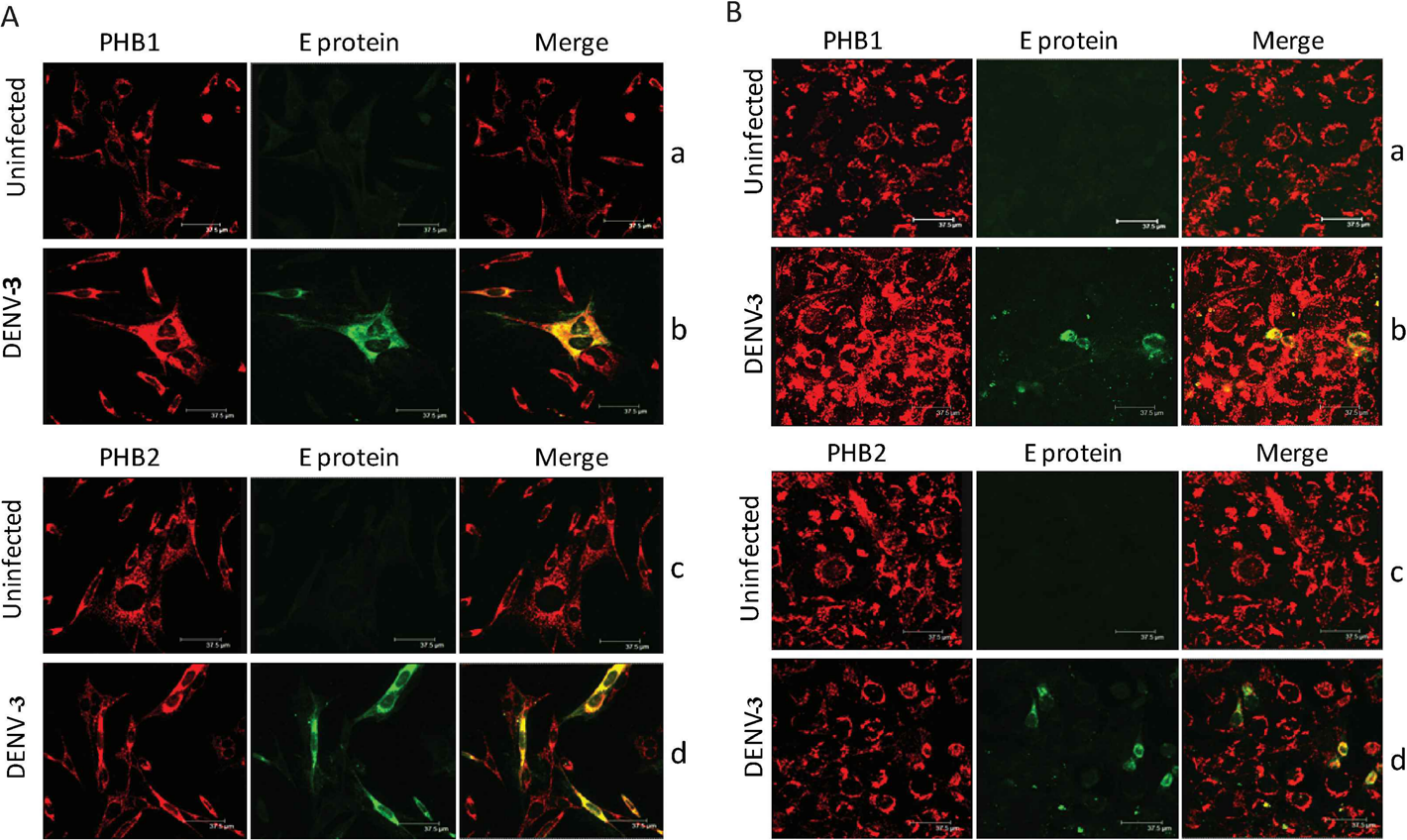
Co-localization of PHB1/2 and DENV-3 infected SH-SY5Y and CHME-3 cells. Representative confocal images of uninfected and DENV-3 infected **A** SH-SY5Y cells and **B** CHME-3 cells, Panel a and b represents cells stained for PHB1 while Panel c and d represents cells stained for PHB2. Merged images represent double labelling of cellular PHB1 (Panel b) and PHB2 (Panel d) with DENV-3 indicating the co-localization (yellow/orange) of PHB1/2 and E-protein of DENV-3

The interaction between PHB1/2 and DENV-3 protein was demonstrated by Co-IP assay. The presence of DENV-3 E protein (45 kDa) in the complex (SH-SY5Y/ CHME-3 membrane + DENV-3, incubated with anti PHB1/2 antibody) was confirmed by Western blot analysis using anti-DENV antibody (Fig. 7, Panel A). Similarly, PHB1/2 protein (30/ 33 kDa) present in the complex (SH-SY5Y/ CHME-3 membrane + DENV-3, incubated with anti-DENV-3 antibody) was confirmed by Western blot analysis using anti PHB1/2 antibody (Fig. 7, Panel B). DENV-3 exhibited co-localization with PHB1/2 in the SH-SY5Y and CHME-3 cells in the double labelling assay (Fig. 8).

### Surface expression of PHB1/2 cells by flow cytometry

**Fig. 9:**
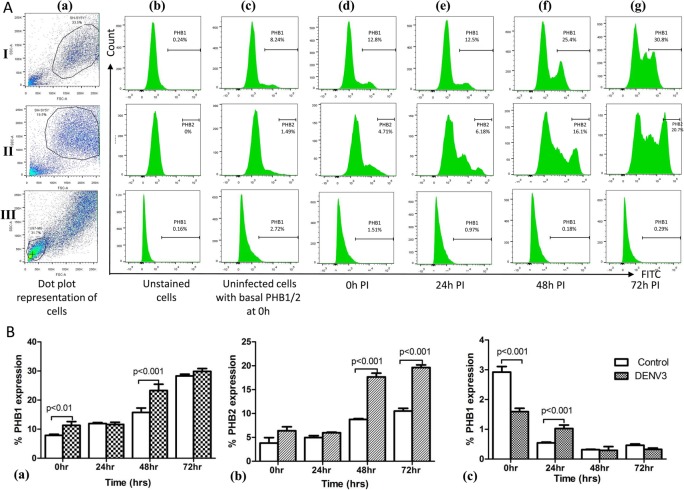
Cell surface expression of PHB1/2 on SH-SY5Y and U-87 MG cells analyzed by Flow cytometry. **A** For expression of PHB1 (Panel I) and PHB2 (Panel II) on SH-SY5Y cells and PHB1 on U-87 MG (Panel III) cells, they were either mock infected (c: representative uninfected control at 0 h) or infected with 10 MOI of DENV-3 and analyzed at 0 h (d), 24 h (e), 48 h (f) and 72 h PI (g). **B** Represents % expression of PHB1/2 on SH-SY5Y (a,b) and U-87 MG (c) cell surface at different time points in comparison to controls. Experiments were performed in triplicate and the error bars represents mean ± SD. Note that PHB1/2 cell surface expression gradually increased at 48 h PI compared to uninfected cells

Flow cytometry was performed with uninfected and DENV-3 infected SH-SY5Y cells to determine the expression of PHB1/2 on the cell surface. SH-SY5Y cell surface expression of PHB1/2 showed an increase in expression at 0 h and 48 h PI (Fig. 9A) with a substantial increase (*p* < 0.001) in infected cells compared to uninfected cells at 48 h PI (Fig. 9B). On the other hand, surface expression of PHB1 on U-87 MG cells exhibited gradual decrease in expression pattern (Fig. 9B). A 62% (PHB1) and 67% (PHB2) increase in expression was observed from 0 h to 72 h PI in SH-SY5Y infected cells. As PHB1 surface expression was not present on U-87 MG cells, it was taken as negative control for PHB1.

### Involvement of PHB1/2 in DENV-3 infection

**Fig. 10:**
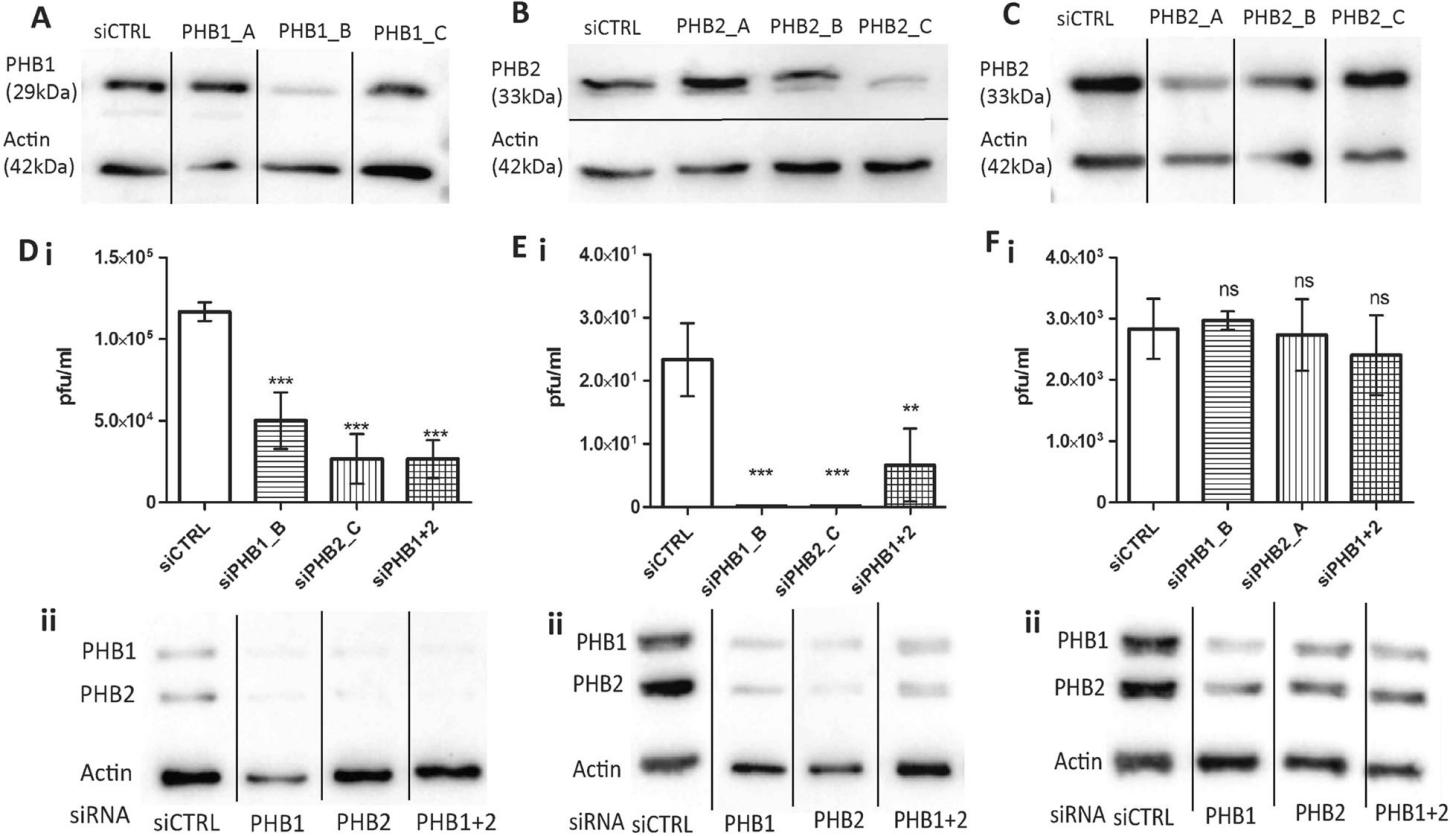
Knockdown of PHB1/2 in DENV-3 infected cells. The efficiency of siRNA knockdown in reverse transfected **A** SH-SY5Y cells with siPHB1_ (A/B/C), **B** SH-SY5Y cells with siPHB2_ (A/B/C) and **C** U-87 MG cells with siPHB2_ (A/B/C) for 48 h was verified by Western blot. Non-targeting siRNA served as control. At 48 h post transfection, cells were infected with DENV-3 (MOI 10). Virus titre in culture supernatant of infected **D-i** SH-SY5Y, **E-i** CHME-3 and **F-i** U-87 MG cells was determination by plaque assay at 30 h PI. Data are presented as mean *±* SD from three independent experiments. Statistical analysis was performed using one-way ANOVA with bonferroni post test (ns *p* > 0.5, ** *p* < 0.001, *** *p* < 0.0001). Cell lysates were harvested from infected (D-ii) SH-SY5Y, (E-ii) CHME-3 and (F-ii) U-87 MG cells for detection of the level of PHB1/2 protein expression by Western blot analysis, using β-actin as an internal control

To further confirm the role of PHB1/2 in DENV-3 infection, siRNA mediated gene silencing was performed on SH-SY5Y, CHME-3 and U-87 MG cells targeting PHB1 and PHB2. Among the three unique siRNA duplexes of PHB1/2, siPHB1_B (sequence: rArGrUrCrUrArUrCrArArArUrGrArArArCrUrCrUrUrUrCAT) exhibited better knockdown across all the three cell lines, whereas, in SH-SY5Y and CHME3 cells, siPHB2_C (sequence: rUrCrUrArUrCrUrCrArCrArGrCrUrGrArCrArArCrCrUrUGT) and in U-87 MG cells, siPHB2_A (sequence: rGrCrUrGrGrArCrUrArCrGrArGrGrArArCrGrArGrUrGrUTG) exhibited better knockdown (Fig. 10A, B, C).

siPHB1, siPHB2, or Control siRNA (siCTRL) were delivered and protein knockdown was determined by Western blot analysis of total cell lysate 48 h post transfection. Interestingly, gene silencing with either PHB1 or PHB2 siRNA resulted in decrease in both protein levels compared to siCTRL (Fig. 10D-ii, E-ii, F-ii). PHB1/2 gene silencing in SH-SY5Y and CHME3 cells resulted in a significant reduction in viral titre in the culture supernatant (Fig. 10D-i, E-i). In SH-SY5Y cells, silencing of PHB1_B, PHB2_C and PHB1 + 2 resulted in decrease in DENV-3 plaques by 57, 78 and 78% respectively. Similarly, a reduction of DENV-3 plaques were observed upon silencing of CHME-3 cells with PHB1_B (100%), PHB2_C (100%) and PHB1 + 2 (72%). On the contrary, no reduction in viral titre was observed upon PHB1/2 gene silencing performed in U-87 MG cells (Fig. 10F-i).

**Fig. 11:**
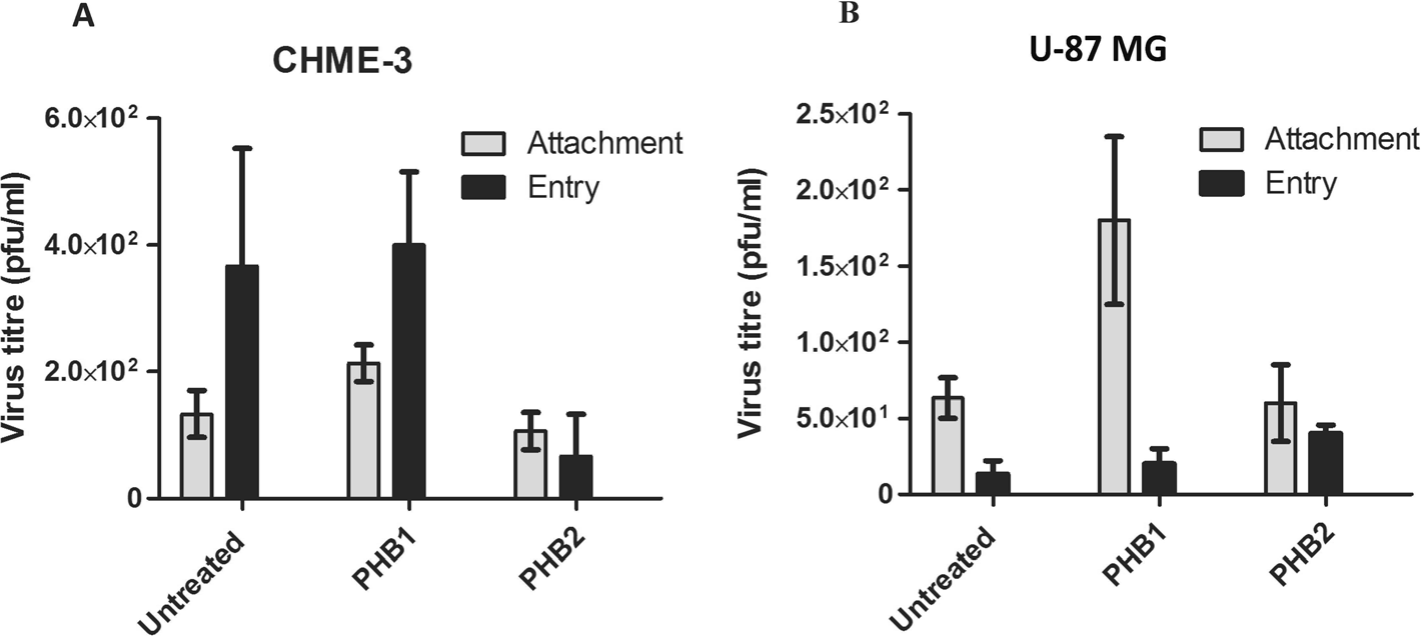
DENV-3 Attachment and Entry assay. Cell culture supernatant from attachment and entry assay was harvested at 30 h PI and plaque assay was performed on LLC-MK2 cells. The virus yield (pfu/ml) obtained in infected **A** CHME-3 cells and **B** U-87 MG cells are presented as mean *±* standard errors of the mean (SEM) from triplicate experiments. Statistics are compared to untreated controls

The attachment and entry assays were carried out to explore the effects of PHB1/2 on DENV-3 binding and entry. The DENV-3 entry into CHME-3 cells treated with PHB2 was decreased compared to untreated cells. PHB2 effectively prevented entry of DENV-3 onto the CHME-3 cells as shown by reduction (82%) in virus titre (Fig. 11A, ‘Entry’: black bars). These results suggest that PHB2 is required for DENV-3 entry into CHME-3 cells. However, no reduction in virus titre was observed in U-87 MG cells with PHB1/2 on attachment or entry.

## Discussion

The initial step in infection requires interaction between the virus and the host cell surface attachment molecules/ receptors which determine the tropism of the virus. In this study, a multifold approach (VOPBA, LC-MS/MS, Co-IP, flow cytometry and siRNA mediated silencing) was employed to identify the molecules on the cell membrane of human neuroblastoma, glioblastoma and microglial cells interacting with DENV-3.

Glycosaminoglycans and sialic acid have been suggested to mediate the attachment and entry of mosquito-borne flaviviruses into mammalian cells [8, 22]. In addition, carbohydrate residues have also been reported to be essential for virus binding to the receptors [23–25]. Interestingly, Chu et al. (2004) identified proteins (55 kDa, 70 kDa, 95 kDa and 140 kDa) implicated in the binding and internalization of WNV, of which two (70 and 95 kDa) were proposed to be part of the receptor complex for mosquito-borne flaviviruses (WNV, JEV and DENV) on C6/36 cells [23]. Earlier studies performed with DENV [8, 10, 14, 26–28] have reported a number of putative cellular receptors such as, glycosaminoglycans, DC-SIGN, laminin receptor, glucose-regulated protein 78 (GRP78), heat shock protein 90 (HSP90), heat shock protein 70 (HSP70) and αVβ3 integrin in different mammalian cell types. Neuronal and glial cells are one of the most commonly affected cells in the brain by viruses. Very few studies have identified receptors for DENV on neural cells. Two earlier studies with DENV-2, have identified a 65 kDa interacting protein [9] and HSP70/ HSP90 as a receptor [10] on human neuroblastoma cells. Brain resident macrophage-like microglia cells are speculated to be targets of DENV infection. Implications of microglial infection with different DENV serotypes [1–4] and identification of the immune mechanisms such as inflammation, regulation, and blood-brain barrier (BBB) breakdown has been made earlier in vitro using murine microglial (BV2) cells [29]. However, there have been no studies identifying the cell surface proteins on neural cells (neurons, astroglia, and microglia) with respect to all DENV serotypes. It is in this context, the present study was undertaken to investigate the targets of DENV-3 serotype in SH-SY5Y, U-87 MG, and CHME-3 cells.

A cell surface molecule is considered as a legitimate virus receptor, if it interacts with viral proteins, it is located on the cell surface, and anti-receptor antibodies or soluble receptor inhibits virus infection. In this study, using contemporary methods such as VOPBA, LC-MS/MS analysis, infection inhibition, surface localization, Co-IP and surface expression, PHB1/2 was identified as a receptor for DENV-3 in SH-SY5Y and CHME-3 cells. A significant reduction in the number of plaques was observed when these cells were treated with anti-PHB2 antibodies on SH-SY5Y (75%) and CHME-3 (82%) cells (Fig. 5). Further, antibodies to PHB1 reduced the infection to 55 and 73% on SH-SY5Y and CHME-3 cells respectively in a dose dependent manner (Fig. 5). Although VIM present on SH-SY5Y cells interacted with DENV-3 in VOPBA; infection inhibition assay using anti-VIM antibodies did not inhibit the virus infection indicating that it is merely an interacting protein. Immunofluorescence assay using anti-PHB2 antibody on non-permeabilized cells revealed localization of PHB2 protein on the cell surface of SH-SY5Y, CHME-3 and U-87 MG cells. On the other hand, PHB1 was detected on the surface of SH-SY5Y and CHME-3 cells but not on U-87 MG cells indicating that both PHB1/2 are required to be present on the cell surface to function as a receptor. The Western blot analysis of the co-immunoprecipitated proteins demonstrated that DENV-3 E protein was an integral part of the complex thereby indicating its active interaction with PHB1 as well as PHB2 on cell surface. Further, double labelling revealed that both PHB1/2 co-localized with DENV-3 E proteins in infected cells. Together these results suggest that PHB1/2 is crucial for DENV-3 attachment /entry into SH-SY5Y and CHME-3 cells and could function as a receptor. However, in U-87 MG cells, no reduction in the number of plaques was observed when the cells were treated with anti-PHB1/2 antibodies. Neither PHB1 nor PHB2 could inhibit DENV-3 entry into U-87 MG cells, indicating that they were merely serving as interacting proteins and not as a receptor.

The role of PHB1/2 was further established by siRNA mediating silencing of PHB1/2 which significantly limited the infection in SH-SY5Y and CHME-3 cells. We observed similar degree of inhibition with both antibodies and siRNA. Complete inhibition of DENV-3 entry into PHB1/2 siRNA silenced cells, suggests that this could be the only pathway used by DENV-3 to enter into CHME-3 cells. Role of PHB in entry and replication has been reported specifically in Enterovirus 71 neuropathogenesis [30].

As PHB1/2 inhibited the DENV-3 infection on CHME-3 cells, the possible mechanism of action could be while attachment or entry process. Our results confirmed that PHB2 is required for viral entry into CHME-3 cells but not into U-87 MG cells (Fig. 11). However, neither PHB1 nor PHB2 blocked viral attachment. This indicated that PHB1/2 function at post attachment level.

Prohibitin is ubiquitously expressed in the cells and is an important member of the membrane protein superfamily. Prohibitin comprises of 2 proteins, with molecular mass of approximately 30 and 37 kDa, and shares about 50% amino-acid identity [31]. They function together as hetero-oligomers which is essential for protein stability, and loss of one prohibitin protein in the cell leads to loss of the other protein [32]. This is controlled at the level of the protein, and is independent of RNA levels [32]. It also plays a role of molecular chaperone in mitochondrial protein stability. Prohibitins are assembled in the inner mitochondrial membrane forming a ring-like structure with alternate PHB1/ 2 subunits [33]. They have been attributed various functions, including cell cycle regulation, apoptosis, assembly of mitochondrial respiratory chain enzymes, and aging. Earlier studies have implicated PHB2 as a receptor for different viruses on various cell types. The interaction between PHB2 and DENV-2 in insect cells has also been documented [32]. Antibody-mediated inhibition of infection and siRNA mediated knockdown of PHB2 expression resulted in significant lowering of virus infection and subsequent virus production in both *A. aegypti* and *A. albopictus* cell lines (CCL-125and C6/36). This indicated that PHB2 can serve as a receptor in insect cell lines. Contrary to this, a subsequent study implicated PHB2 as a refractivity conferring non-receptor molecule for DENV-2 on the *A. aegypti* midgut BBMF proteins, rather than a receptor as proposed by Kuadkitkan et al. [34]. An earlier study identified PHB1 as a receptor for CHIKV on human microglial (CHME-5) cells [35].

A cross reactivity has been observed with the anti-human PHB2 polyclonal antibody detecting both PHB1 and PHB2 of insect origin [32], approximately 75% homology exist between human PHB1/2 and PHB of *A. aegypti*. We observed that silencing of either PHB1 or PHB2, leads to reduction in protein level of both the proteins, which suggests they are interconnected. This is in consistent with the PHB1/2 knockdown of Huh7.5.1 cells [36].

The lateral mobility of most receptor molecules in the cell membrane allows the formation of a local microdomain rich in receptors under the bound virus where the composition and properties differ from that in the surrounding membrane [37]. There is a likelihood of receptor clustering on the cell membrane from 24 h onwards which increased considerably at 48 h and 72 h PI as the infection proceeded [37]. Indeed in the present study, a gradual increase in expression of PHB1/2 was observed in infected SH-SY5Y cells from 24 h PI to 72 h PI by flow cytometric analysis. The dynamic recruitment of fresh PHB1/2 from inner mitochondrial membrane and transport to cell membrane could possibly explain the increase in PHB1/2 level (Fig. 9).

The traditional belief of a virus binding to a single receptor for gaining entry into cells is being replaced by a more complex notion. At least two different receptors are known to be used by several viruses to bind to their host cells: (i) the binding receptors are those that allow the virus to attach to the cell surface quickly, and (ii) those receptors that facilitate post-binding, post-attachment, entry and fusion are referred to as co-receptors. For instance, in the case of HIV-1, HSV 1 and 2, adenovirus and measles virus [38], multiple interactions between the virus and cell surface molecules have been proposed. A prominent example of a dual receptor is observed in HIV-1 binding where CD4 binds to the cell surface and co-receptors such as CXCR4 and CCR5 facilitates infusion of the viral envelope and cell membrane. From the present study, it was evident that DENV-3 required PHB1/2 as a receptor complex for entry into SH-SY5Y and CHME-3 cells. In a more recent study, both VIM and integrin have been shown to act as a direct receptor and co-receptor involved in endocytosis of DENV-2 [39]. Similarly, an earlier study had reported that DENV-2 requires a group of molecules (HSP-90 and HSP-70) as receptor complex to gain entry into human monocytic (U-937) cells and human neuroblastoma (SK-SY-5Y) cells [10]. DENV-4 infection in Vero cells requires heparan sulphate as a primary receptor and a 74 kDa protein as a co-receptor [40]. Therefore, it is very likely that PHB1/2 identified in this study serve as a putative receptor complex for entry of DENV-3 into SH-SY5Y and CHME-3 cells.

## Conclusion

This is the first study to describe receptors for DENV-3 serotype on the surface of neural cells. Although a number of proteins on neural cells interacting with DENV-3 serotype were identified, only PHB2 and PHB1 proteins qualified as putative receptors. It would be interesting to confirm if the same proteins also serve as receptors on primary neural cells of human origin.

## Data Availability

The datasets used and/or analysed during the current study are available from the corresponding author on reasonable request.

## Abbreviations

DENV: Dengue virus
PHB1/2: Prohibitin1/2
FBS: Fetal bovine serum
pAb: Polyclonal antibody
VDAC: Voltage-dependent anion channel
PBS: Phosphate-buffered saline
VOPBA: Virus overlay protein binding assay
LC-MS/MS: Liquid chromatography-mass spectrometry
ABC: Ammonium bicarbonate (ABC)
ACN: Acetonitrile
DTT: Dithiothreitol
VIM: Vimentin
PI: Post infection
